# Epidemiology, outcomes and predictors of mortality in patients transported by ambulance for dyspnoea: A population‐based cohort study

**DOI:** 10.1111/1742-6723.14053

**Published:** 2022-08-02

**Authors:** Jennifer Zhou, Emily Nehme, Luke Dawson, Jason Bloom, Ziad Nehme, Daniel Okyere, Shelley Cox, David Anderson, Michael Stephenson, Karen Smith, David M Kaye, Dion Stub

**Affiliations:** ^1^ Department of Cardiology Alfred Health Melbourne Victoria Australia; ^2^ Ambulance Victoria Melbourne Victoria Australia; ^3^ School of Public Health and Preventive Medicine Monash University Melbourne Victoria Australia; ^4^ Department of Paramedicine Monash University Melbourne Victoria Australia; ^5^ Baker Heart and Diabetes Institute Melbourne Victoria Australia

**Keywords:** ambulance, dyspnoea, emergency medicine, epidemiology, shortness of breath

## Abstract

**Objectives:**

There are currently limited data to inform the management of patients transported by emergency medical services (EMS) with dyspnoea. We aimed to describe the incidence, aetiology and outcomes of patients transported by EMS for dyspnoea using a large population‐based sample and to identify factors associated with 30‐day mortality.

**Methods:**

Consecutive EMS attendances for dyspnoea in Victoria, Australia from January 2015 to June 2019 were included. Data were individually linked to hospital and mortality records to determine incidence, diagnoses, and outcomes. Factors associated with 30‐day mortality were assessed using multivariable logistic regression.

**Results:**

During the study period, there were 2 505 324 cases attended by EMS, of whom 346 228 (14%) met inclusion criteria for dyspnoea. The incidence of EMS attendances for dyspnoea was 1566 per 100 000 person‐years, and was higher in females, older patients and socially disadvantaged areas. Of the 271 204 successfully linked cases (median age 76 years; 51% women), 79% required hospital admission with a 30‐day mortality of 9%. The most common final diagnoses (and 30‐day mortality rates) were lower respiratory tract infection (13%, mortality 11%), chronic obstructive pulmonary disease (13%, mortality 6.4%), heart failure (9.1%, mortality 9.8%), arrhythmias (3.9%, mortality 4.4%), acute coronary syndromes (3.9%, mortality 9.5%) and asthma (3.2%, mortality 0.5%). Predictors of mortality included older age, male sex, pre‐existing chronic kidney disease, heart failure or cancer, abnormal respiratory status or vital signs and pre‐hospital intubation.

**Conclusion:**

Dyspnoea is a common presentation with a broad range of causes and is associated with high rates of hospitalisation and death.

## Graphical abstract

We performed a large retrospective population‐based cohort study of all emergency medical services (EMS) attendances for dyspnoea in Victoria, Australia over a 4.5‐year period. We found that dyspnoea is a common symptom accounting for one in seven EMS attendances, and more commonly affects females, the elderly and areas of socioeconomic disadvantage. Patients transported by EMS with dyspnoea have a high morbidity and mortality, with over three quarters admitted to hospital and a 9% mortality rate at 30 days.

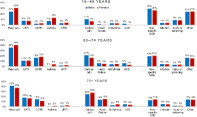

**Key findings**

- Dyspnoea is a common symptom accounting for one in seven EMS attendances in Victoria, with a higher burden in females, the elderly and in areas of socioeconomic disadvantage.
- Patients transported by EMS with dyspnoea have a high morbidity and mortality, with over three quarters admitted to hospital and a 30‐day mortality of 9.0%.
- Predictors of 30‐day mortality included older age, male sex, pre‐existing CKD, heart failure or cancer, abnormal respiratory status, abnormal vital signs and pre‐hospital intubation.

## Introduction

Dyspnoea is a common symptom accounting for over 10% of hospital admissions in Australia and is associated with substantial morbidity and mortality.^1^, ^2^ The causes for dyspnoea are varied and range from benign to life‐threatening. As such, patients with dyspnoea often pose a diagnostic and management challenge, as clinicians must work through a broad range of differential diagnoses while initiating therapy for potentially time critical conditions.

Emergency medical services (EMS) play an important role in the triage, transport, and initial management of patients with dyspnoea. Studies have shown that the prognosis of patients with breathlessness corresponds to the accuracy of initial assessment.^3^ Despite this, little is known about the epidemiology and outcomes of undifferentiated patients attended by paramedics for dyspnoea.

In the present study, we used a large population‐based ambulance database linked with emergency, hospital admission and mortality records to describe the incidence, aetiology and outcomes of patients managed by paramedics for dyspnoea, and to identify variables associated with 30‐day mortality.

## Methods

This was a retrospective cohort study of all EMS encounters for adults with dyspnoea between 1 January 2015 and 30 June 2019 in Victoria, Australia. Ethics approval was granted by the Monash University Human Research Ethics Committee (number 11681).

### Data sources and setting

Ambulance Victoria is the sole EMS provider to the state of Victoria, servicing a land area of approximately 227 444 km^2^ and a population of 6.7 million people.^4^ All cases attended by paramedics are documented in an electronic patient care record and uploaded to Ambulance Victoria's data warehouse.

In the present study, we used data linkage to combine Ambulance Victoria patient care record data with other relevant datasets: (i) VEMD – a Victorian Department of Health administrative and clinical dataset including all ED presentations at public hospitals in the state; (ii) VAED – a Victorian Department of Health demographic, clinical and administrative dataset detailing each admitted episode of care occurring in public and private hospitals, as well as rehabilitation centres, extended care facilities and day procedure centres in the state; and (iii) VDI – a Victorian Department of Health dataset that captures the date and cause of all deaths in Victoria. Linkage processes have been described previously and are detailed in Appendix S1.^5^

### Inclusion and exclusion criteria

Criteria for inclusion in the study were: (i) dyspnoea or shortness of breath documented in the clinical records, or (ii) a final or secondary ambulance diagnosis of shortness of breath, respiratory distress, heart failure or airways disease, or (iii) initial respiratory status documented as mild, moderate or severe respiratory distress, apnoeic or ventilated. Exclusion criteria included: (i) attendances recorded as having a case nature of ‘Trauma’, (ii) ambulance attendances for transfers between hospitals, (iii) age less than 18 years, and (iv) out‐of‐hospital cardiac arrest prior to ambulance arrival.

### Outcomes

The primary endpoint was mortality at 30 days. Other endpoints included pre‐hospital characteristics, final diagnoses (defined using International Classification of Disease 10 codes as the VAED primary diagnosis if discharged from hospital or the VEMD primary diagnosis if discharged from ED) and rates of non‐invasive ventilation (NIV), mechanical ventilation, hospital and intensive care (ICU) admission. Finally, we sought to identify factors predictive of 30‐day mortality.

### Statistical methods

Categorical variables were presented as number (percentage) and continuous variables were presented as mean (standard deviation [SD]) or median (interquartile range [IQR]). Standardised differences were used to examine differences between patient groups. Incidence rates were calculated using age and sex‐specific population estimates available from the Australian Bureau of Statistics (Fig. S1). 95% confidence intervals (CIs) for incidence rates were calculated assuming a Poisson distribution. The Cochrane‐Armitage test was used to assess for changes in incidence over time.

Univariable logistic regression was used to identify factors associated with 30‐day mortality. Co‐variates of interest included: age, sex, remoteness, socioeconomic status, comorbidities (hypertension, dyslipidaemia, diabetes, coronary artery disease [CAD], heart failure, chronic obstructive pulmonary disease [COPD], chronic kidney disease [CKD] and cancer), initial respiratory status, initial vital signs (hypotension [systolic blood pressure <90 mmHg], hypertension [systolic blood pressure >180 mmHg], tachycardia [heart rate ≥100 bpm], tachypnoea [respiratory rate >20 breaths per minute], bradypnoea [respiratory rate <12 breaths per minute], hypoxia [oxygen saturations <90%] and fever [temperature ≥38.0°C]), and pre‐hospital NIV or intubation. Variables with a *P*‐value <0.10 in the univariable regression were included in multivariable models. Models were adjusted for final diagnosis.

Data were analysed using Stata version 15.1 for Windows (College Station, TX, USA). All *P*‐values were two‐sided and a *P*‐value <0.05 was considered statistically significant.

## Results

Between January 2015 and June 2019 (representing 22 112 096 person‐years), there were 2 505 324 unique ambulance attendances in Victoria. Of these, one in seven cases (*n* = 346 228) met inclusion criteria for dyspnoea. The population‐wide incidence of EMS attendances for dyspnoea was 1566 per 100 000 person‐years (95% CI 1561–1571) with a higher incidence in females, older patients and areas of socioeconomic disadvantage (Table S1). The incidence of EMS attendances for dyspnoea increased over time (1552 *vs* 1594 per 100 000 person‐years in 2015 *vs* 2019, *p*_for trend_ = 0.009).

Of the total attendances, 620 patients (0.2%) died at the scene, 178 (0.05%) received palliative treatment, 9716 (2.8%) refused transport, and 17 687 (5.1%) were deemed by paramedics not to require transport. Compared to those transported, patients deemed not to require transport were younger, had fewer comorbidities, and were more likely to have normal vital signs. The 30‐day mortality for patients not transported by EMS was 3.1% compared to 8.1% in those transported (Table S2).

Of the transported cohort, 271 204 patients (85%) were successfully linked to VAED or VEMD databases and were included in the final analysis. Differences between linked and unlinked cases are presented in Table S3.

### Patient characteristics

Characteristics of the linked cohort are presented in Table 1. The median age was 74 years (IQR 59–83 years) and 51.4% were female. There was a high burden of comorbidities. Approximately half the patients had either mild, moderate or severe respiratory distress. Most other patients had normal respiratory status, while a small proportion had depressed ventilations (0.1%) or were apnoeic (0.2%). The median initial respiratory rate was 24 breaths per minute (IQR 18–28) and the median oxygen saturation was 95% (IQR 90–98).

**TABLE 1:** Baseline characteristics of linked cohort

	18–49 years	50–74 years	75+ years	Overall
	*n* = 42 638	*n* = 98 119	*n* = 130 402	*n* = 271 204
Age (years)	38 (29–45)	65 (59–70)	84 (79–88)	74 (59–83)
Female sex	24 937 (58.5%)	46 619 (47.5%)	67 749 (52.0%)	139 305 (51.4%)
ARIA				
Major city	31 805 (75.9%)	68 522 (70.2%)	98 087 (75.4%)	198 414 (73.6%)
Inner regional	8436 (20.1%)	23 661 (24.3%)	25 987 (20.0%)	58 084 (21.5%)
Outer regional	1675 (3.9%)	5386 (5.5%)	6057 (4.7%)	13 118 (4.8%)
SES				
Quintile 1 (lowest)	11 150 (29.7%)	28 902 (32.2%)	32 505 (27.2%)	72 557 (29.4%)
Quintile 2	7914 (21.1%)	20 188 (22.5%)	26 427 (22.1%)	54 529 (22.1%)
Quintile 3	7244 (19.3%)	17 004 (19.0%)	24 128 (20.2%)	48 376 (19.6%)
Quintile 4	6746 (18.0%)	14 016 (15.6%)	21 433 (18.0%)	42 195 (17.1%)
Quintile 5 (highest)	4479 (11.9%)	9587 (10.7%)	14 869 (12.5%)	28 835 (11.7%)
Comorbidities				
Hypertension	4278 (10.3%)	40 154 (41.2%)	72 206 (55.7%)	116 638 (43.4%)
Dyslipidaemia	2448 (5.7%)	27 543 (28.3%)	43 234 (33.3%)	73 225 (27.2%)
Diabetes	3655 (8.6%)	23 581 (24.2%)	32 580 (25.1%)	59 816 (22.2%)
Coronary artery disease	2227 (5.2%)	23 456 (24.1%)	32 968 (33.1%)	68 651 (25.5%)
Heart failure	656 (1.6%)	10 361 (10.6%)	34 715 (26.8%)	45 732 (17.0%)
Chronic kidney disease	658 (1.5%)	4677 (4.8%)	10 260 (7.9%)	15 595 (5.8%)
Atrial fibrillation	607 (1.4%)	10 313 (10.6%)	31 203 (24.1%)	42 123 (15.7%)
Cancer	1636 (3.8%)	15 000 (15.4%)	21 376 (16.5%)	38 012 (14.1%)
COPD	1991 (4.8%)	27 251 (28.0%)	34 583 (26.7%)	63 825 (23.7%)
Respiratory status				
Normal	19 751 (55.2%)	38 429 (47.5%)	44 589 (41.8%)	102 769 (46.0%)
Mild respiratory distress	9515 (26.6%)	22 350 (27.6%)	35 696 (33.5%)	67 561 (30.2%)
Moderate respiratory distress	3886 (10.9%)	12 811 (15.8%)	17 784 (16.7%)	34 481 (15.4%)
Severe respiratory distress	2137 (5.0%)	7077 (8.7%)	8376 (8.0%)	17 590 (6.5%)
Respiratory depression	119 (0.3%)	98 (0.1%)	92 (0.1%)	309 (0.1%)
Apnoeic	169 (0.5%)	97 (0.1%)	60 (0.1%)	326 (0.2%)
Clinical features on initial paramedic assessment				
Respiratory rate	20 (18–28)	22 (18–28)	24 (20–30)	24 (18–28)
SpO_2_ (%)	98 (95–99)	95 (90–98)	94 (89–97)	95 (90–98)
Febrile (*T* ≥38.0)	4655 (11.9%)	14 307 (15.4%)	22 634 (18.1%)	41 596 (16.2%)
Tachycardic (HR ≥100)	23 642 (55.6%)	45 425 (46.4%)	46 802 (36.0%)	115 869 (42.9%)
Hypotensive (SBP <90)	1179 (2.8%)	3189 (3.2%)	4185 (3.2%)	8553 (3.2%)
Hypertensive (SBP >180)	834 (2.0%)	6392 (6.6%)	11 445 (8.8%)	18 671 (6.9%)

Data are provided as number (percentage) or median (interquartile range). ARIA, Accessibility/Remoteness Index of Australia; COPD, chronic obstructive pulmonary disease; HR, heart rate; SBP, systolic blood pressure; SES, socioeconomic status; SpO_2_, oxygen saturations; *T*, temperature.

### Pre‐hospital care

EMS providers determined the illness severity as time‐critical in 14% and urgent in 80% of cases (Table S4). The median time to arrival of paramedics was 11 min (IQR 8–18 min) and the median total case time was 105 min (IQR 86–128 min). Most patients (93%) were transported to public hospitals.

Pre‐hospital oxygen therapy was administered in 39.5% of patients, while 2.3% received NIV and 0.3% were intubated by paramedics. Other common pre‐hospital interventions included inhaled bronchodilators (17.3%), corticosteroids (5.5%), aspirin (11.6%), glyceryl trinitrate (14.6%) and intravenous fluids (9.1%). Antibiotic therapy was used rarely (0.04% of cases).

Pre‐hospital interventions, stratified by final hospital diagnosis, are presented in Table S5. Bronchospasm appeared to be most readily identified diagnosis by paramedics, with over 60% of those eventually diagnosed with asthma or COPD treated with inhaled bronchodilators in the pre‐hospital setting.

### Hospital management

Most patients (79.0%) transported by EMS were admitted to hospital. Patients admitted to hospital were older and more likely to have comorbidities or abnormal vital signs compared to those who were discharged directly from the ED (Table S6). The median hospital length of stay was 3 days (IQR 1–6 days). NIV was utilised in 6.6% of patients during their hospital stay, and 1.5% of the cohort required mechanical ventilation. 11.2% of patients were admitted to ICU. For these patients, the median time spent in the ICU was 50 h (IQR 25–93 h).

### Final diagnoses

**Figure 1:**
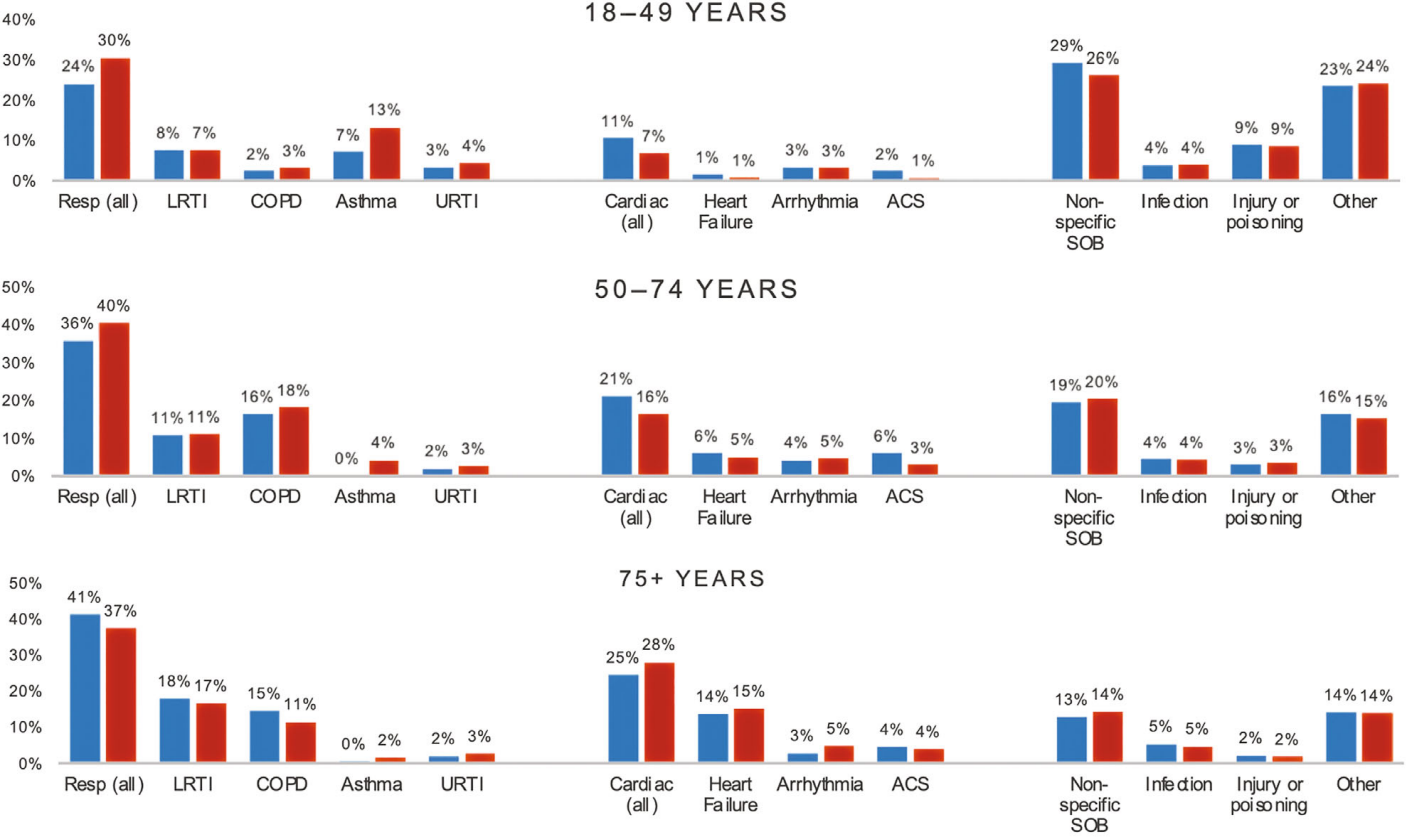
Final hospital diagnoses by age and sex. The percentage of patients with each final diagnosis are presented for each age group (18–49 years upper panel, 50–74 years middle panel and 75+ years lower panel), stratified by sex (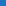 indicates male, 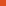 indicates female). ACS, acute coronary syndromes; COPD, chronic obstructive pulmonary disease; LRTI, lower respiratory tract infection; SOB, shortness of breath; URTI, upper respiratory tract infection.

Final diagnoses, stratified by age group and sex, are presented in Figure 1 and Table S7. The most common final diagnostic groups were respiratory (37.0%), followed by cardiovascular (20.8%), infection (4.5%), poisoning or injury (3.7%), gastrointestinal (3.4%), oncological (2.8%), mental health (2.1%), rheumatological (1.5%), endocrinological (1.5%) and neurological (0.9%) disorders. The most common specific diagnoses were lower respiratory tract infection (13.4%), COPD (12.9%), heart failure (9.1%), asthma (3.2%), arrhythmias (3.9%) and acute coronary syndromes (non‐ST elevation acute coronary syndrome [NSTEACS] 3.1% and ST elevation myocardial infarction [STEMI] 0.8%). 18.0% of cases were coded as non‐specific dyspnoea.

The distribution of final diagnoses varied by age. In those over 75 years, most diagnoses were either respiratory or cardiovascular. In contrast, cardiorespiratory conditions formed a lower proportion of diagnoses in the 18 to 49 years age group, and non‐specific dyspnoea, poisoning or injury, and mental health diagnoses were more frequently encountered.

### Mortality

**Figure 2:**
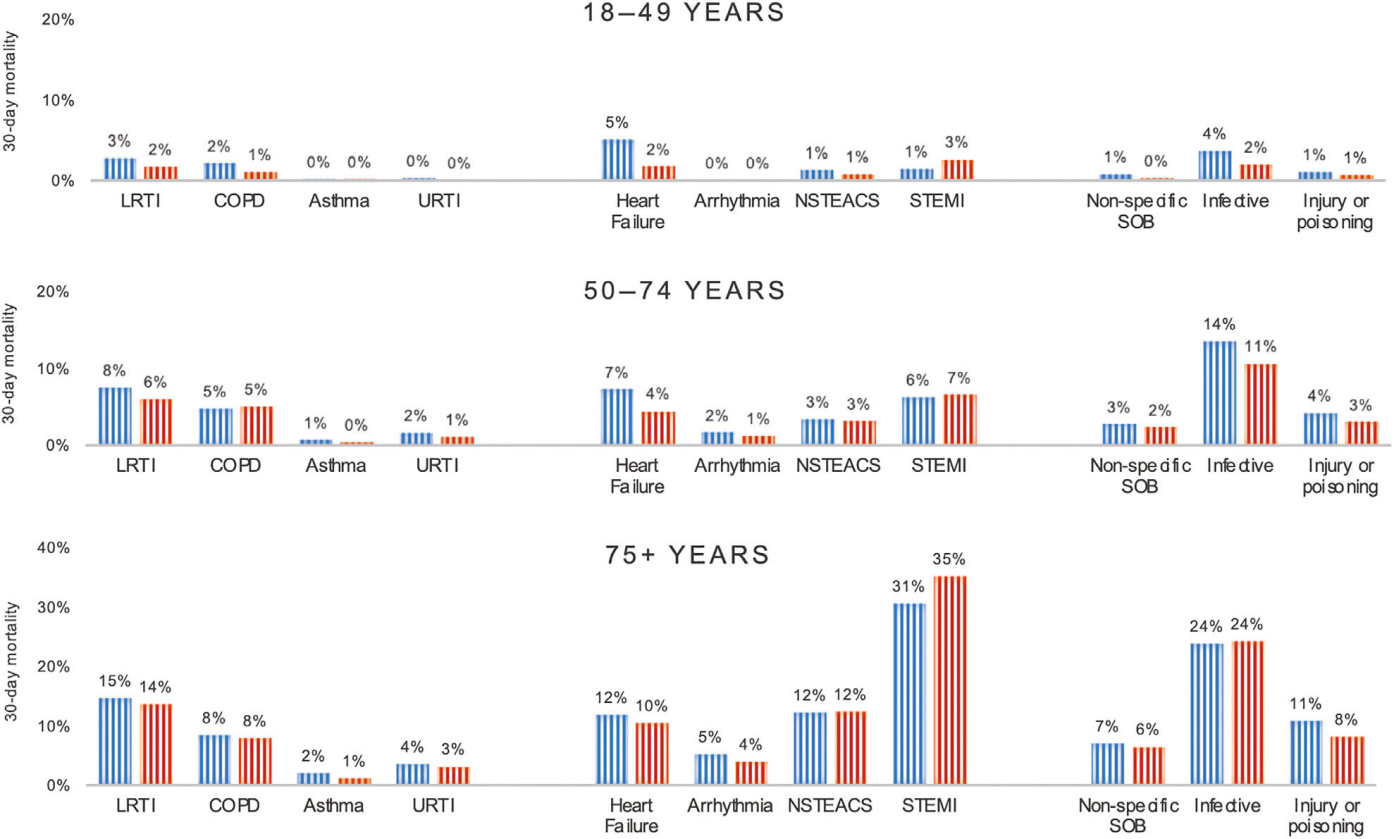
Thirty‐day mortality for the most common final diagnoses, by age and sex. Thirty‐day mortality rates for each of the most common final diagnoses are presented for each age group (18–49 years upper panel, 50–74 years middle panel and 75+ years lower panel) stratified by sex (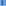 indicates male, 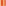 indicates female). COPD, chronic obstructive pulmonary disease; LRTI, lower respiratory tract infection; NSTEACS, non ST‐elevation myocardial infarction; STEMI, ST elevation myocardial infarction; URTI, upper respiratory tract infection.

Thirty‐day mortality rates are presented in Figure 2, Tables S8 and S9. Overall, 9.0% of patients were deceased at 30 days. Patients discharged directly from the ED had better outcomes (30‐day mortality 2.3%) than those admitted to hospital (30‐day mortality 9.6%) or to ICU (30‐day mortality 12.7%).

Among the most common specific diagnoses, STEMI had the worst prognosis with a 30‐day mortality of 14.3%, followed by 10.9% for lower respiratory tract infection, 9.8% for heart failure, 8.3% for NSTEACS, 6.9% for non‐AF arrhythmias, 6.4% for COPD, 3.6% for non‐specific dyspnoea, 2.9% for AF and 0.5% for asthma. The diagnoses with the worst prognoses were oncological disorders (30‐day mortality 36.2%), sepsis (30‐day mortality 25.8%) and interstitial lung disease (30‐day mortality 21.8%). In most diagnosis categories, females had a lower 30‐day mortality compared to males.

### Pre‐hospital predictors of mortality

Factors associated with 30‐day mortality in the multivariable logistic regression, adjusted for final diagnosis, are presented in Table 2. Demographic factors independently predictive of 30‐day mortality included older age, male sex and pre‐existing CKD, heart failure or cancer. Clinical predictors of 30‐day mortality were abnormal respiratory status, abnormal vital signs (tachypnoea, bradypnoea, hypoxia, tachycardia or hypotension) and pre‐hospital intubation.

**TABLE 2:** Pre‐hospital predictors of 30‐day mortality

Variable	Univariable analysis (OR)	*P*‐value	Multivariable analysis (OR)	*P*‐value
Age (years)				
<50	1 [Reference]		1 [Reference]	
50–59	2.79 (2.54–3.07)	<0.001	2.69 (2.40–3.03)	<0.001
60–69	4.42 (4.07–4.81)	<0.001	3.76 (3.39–4.17)	<0.001
70–79	6.03 (5.57–6.53)	<0.001	5.33 (4.82–5.89)	<0.001
80+	10.01 (9.31–10.86)	<0.001	9.47 (8.59–10.43)	<0.001
Male sex	1.23 (1.20–1.26)	<0.001	1.12 (1.08–1.16)	<0.001
Remoteness				
Metropolitan	1 [Reference]		†	†
Remote or regional	0.93 (0.90–0.96)	<0.001	†	†
Socioeconomic status			†	
Quintile 1 (lowest)	1 [Reference]		†	†
Quintile 2	1.04 (0.97–1.08)	0.076	†	†
Quintile 3	0.99 (0.96–1.04)	0.996	†	†
Quintile 4	1.01 (0.97–1.06)	0.584	†	†
Quintile 5 (highest)	1.09 (1.04–1.13)	0.001	†	†
Comorbidities				
Hypertension	0.99 (0.97–1.02)	0.709	†	†
Hypercholesteraemia	0.75 (0.73–0.77)	<0.001	0.66 (0.64–0.69)	<0.001
Diabetes mellitus	1.12 (1.08–1.15)	<0.001	†	†
Chronic kidney disease	1.84 (1.76–1.93)	<0.001	1.59 (1.50–1.68)	<0.001
Coronary artery disease	1.06 (1.03–1.09)	<0.001	0.96 (0.92–0.99)	0.03
Heart failure	1.76 (1.70–1.81)	<0.001	1.33 (1.28–1.39)	<0.001
COPD	1.16 (1.13–1.95)	<0.001	0.92 (0.89–0.96)	0.001
Cancer	2.83 (2.84–3.02)	<0.001	2.05 (1.97–2.13)	<0.001
Initial respiratory status				
Normal respiratory status	1 [Reference]		1 [Reference]	
Mild respiratory distress	2.07 (1.99–2.15)	<0.001	1.44 (1.37–1.50)	<0.001
Moderate respiratory distress	2.87 (2.75–2.99)	<0.001	1.82 (1.73–1.92)	<0.001
Severe respiratory distress	4.34 (4.14–4.54)	<0.001	2.67 (2.50–2.85)	<0.001
Depressed ventilation	8.36 (6.55–10.66)	<0.001	4.85 (3.48–6.76)	<0.001
Apnoeic	4.87 (3.72–6.38)	<0.001	2.95 (1.98–4.42)	<0.001
Initial vital signs				
Hypoxia (SpO_2_ <90%)	3.37 (3.28–3.46)	<0.001	1.97 (1.89–2.04)	<0.001
Tachypnoea (RR >20)	2.37 (2.29–2.43)	<0.001	1.41 (1.34–1.47)	<0.001
Bradypnoea (RR <12)	2.09 (1.82–2.41)	<0.001	1.30 (1.03–1.65)	0.01
Tachycardia (HR ≥100)	1.51 (1.47–1.55)	<0.001	1.40 (1.34–1.47)	<0.001
Hypotension (SBP <90)	4.37 (4.16–4.59)	<0.001	2.87 (2.69–3.07)	<0.001
Hypertension (SBP >180)	0.56 (0.53–0.61)	<0.001	0.46 (0.43–0.50)	<0.001
Fever (*T* ≥38.0)	1.07 (1.04–1.11)	<0.001	0.60 (0.58–0.64)	<0.001
Pre‐hospital management				
Non‐invasive ventilation	2.00 (1.87–2.15)	<0.001	0.86 (0.79–0.95)	0.001
Intubation	8.33 (7.24–9.59)	<0.001	7.45 (6.15–9.04)	<0.001

† Not significant in multivariable logistic regression so not included in final model.

COPD, chronic obstructive pulmonary disease; HR, heart rate; OR, odds ratio; RR, respiratory rate; SBP, systolic blood pressure; SpO_2_, oxygen saturations; *T*, temperature.

Factors associated with a reduced risk of 30‐day mortality included female sex, pre‐existing hypercholesteraemia, coronary artery disease or COPD, and fever or hypertension. Use of pre‐hospital NIV, compared with no ventilatory support, when adjusted for age, sex, comorbidities, respiratory status and vital signs, was also associated with a reduced risk of 30‐day mortality.

## Discussion

In this population‐based cohort study, we assessed the incidence, aetiology and outcomes of patients attended by ambulance for dyspnoea in Victoria, Australia over a 4.5‐year period, and evaluated factors associated with 30‐day mortality. Through linkage of four state‐wide registries, our study provides unique insight into the burden, causes and outcomes of patients attended by EMS for dyspnoea, and may have implications for service planning, training and future research in this field.

Our findings can be summarised as follows: (i) dyspnoea is a common symptom accounting for one in seven EMS attendances in Victoria, with a higher burden in females, the elderly and in areas of socioeconomic disadvantage; (ii) patients transported by EMS with dyspnoea have a high morbidity and mortality, with over three quarters admitted to hospital and a 30‐day mortality of 9.0%; (iii) the causes for dyspnoea are varied and range from benign to life‐threatening; and (iv) predictors of 30‐day mortality included older age, male sex, pre‐existing CKD, heart failure or cancer, abnormal respiratory status, abnormal vital signs and pre‐hospital intubation.

While several previous studies have examined the epidemiology of patients presenting with dyspnoea, these have generally been smaller^6^, ^7^ or have been limited to inpatient or ED settings.^1^, ^8^, ^9^ Our study is the largest to date to describe the incidence and outcomes of patients requiring emergency care for dyspnoea and highlights the high and increasing burden of disease. In contrast to prior studies, we also specifically focus on the pre‐hospital setting, a uniquely challenging environment where presentations are often undifferentiated, and paramedic treatment decisions must often rely on clinical judgement and a knowledge of disease patterns in the population.

The most common diagnoses in our study were respiratory and cardiovascular, specifically lower respiratory tract infection, COPD, heart failure, arrhythmias, acute coronary syndromes and asthma. However, non‐cardiac and non‐respiratory conditions were also common, and in younger patients formed the bulk of final diagnoses. The diverse range of causes for breathlessness has frequently been identified as a major challenge in treating patients with this symptom. Strategies to better delineate the causes of dyspnoea in the pre‐hospital setting, and to identify those at risk of adverse outcomes, are urgently required.

Through multivariable analyses, we identified several factors predictive of 30‐day mortality in our cohort. These included demographic factors such as older age, male sex and pre‐existing CKD, heart failure or cancer, and clinical factors such as abnormal vital signs, abnormal respiratory status and pre‐hospital intubation. In future, development of decision tools that incorporate readily available demographic and clinical factors may be helpful to risk stratify patients and guide resource allocation in the pre‐hospital setting.

Current patterns in the pre‐hospital management of patients with dyspnoea were also highlighted in our study. Inhaled bronchodilators were frequently used, corresponding to high rates of asthma and COPD, and perhaps the more ready identification of these disorders by paramedics. Cardiac interventions such as aspirin and nitrates were also common, reflecting well‐established protocols for patients with suspected ACS. Notably, antibiotics were used in only 0.04% of cases, despite high rates of lower respiratory tract infection and sepsis. This may be because antibiotics are not currently a routine part of Ambulance Victoria treatment protocols for sepsis.^10^ This may represent an unmet opportunity to improve pre‐hospital care, particularly with growing data to support early antibiotic administration in sepsis.^11^

Finally, despite data indicating that use of NIV in patients with respiratory distress reduces the need for intubation and improves survival, only 2.2% of patients were treated with pre‐hospital NIV in our study.^12^ In multivariable analyses, pre‐hospital use of NIV was associated with a lower 30‐day mortality when adjusted for patient demographics, comorbidities, clinical factors and diagnosis. While these data should be recognised as exploratory, it is possible that the pre‐hospital application of NIV in patients with respiratory distress may represent a target to improve outcomes. Further prospective studies are required to evaluate the feasibility and safety of this.

### Study limitations

Several limitations of our study warrant mention. For one, our study only included patients who were transported by EMS for dyspnoea and did not include those who self‐presented to hospital or who developed dyspnoea after arrival to hospital. Thus, our findings are specific to patients attended by EMS for dyspnoea and may not be applicable to all patients with shortness of breath. Second, there were limited data available regarding in‐hospital interventions. Finally, a significant proportion of patients were not successfully linked to the VAED and VEMD databases and were therefore not included in the primary analysis. This could represent a source of selection bias.

## Conclusion

Dyspnoea accounts for one in seven EMS attendances in Victoria, Australia. It more commonly affects females, the elderly and areas of socioeconomic disadvantage, and has a high morbidity and mortality. Priorities for future research include the development of decision tools to delineate between the diverse causes of dyspnoea and to risk stratify patients in the pre‐hospital setting.

## Supporting information


**Appendix S1**. Dataset linkage processes.

**Figure S1**. Cohort derivation.

**Table S1**. Incidence of shortness of breath.

**Table S2**. Differences in patient characteristics between those deemed by paramedics not to require transport and those transported to hospital.

**Table S3**. Differences in patient characteristics between the linked and unlinked cohorts.

**Table S4**. Ambulance and hospital management details.

**Table S5**. Pre‐hospital interventions administered by EMS and concordance with final hospital diagnosis.

**Table S6**. Differences in patient characteristics between those discharged versus admitted from the ED.

**Table S7**. Final hospital diagnoses stratified by sex and age group.

**Table S8**. Mortality among patients transported by EMS.

**Table S9**. 30‐day mortality rates for each diagnosis stratified by sex and age group.

## Data Availability

The data that support the findings of the present study are available from the corresponding author upon reasonable request.

## References

[1] Kelly AM , Keijzers G , Klim S *et al*. An observational study of dyspnea in emergency departments: the Asia, Australia, and New Zealand dyspnea in emergency departments study (AANZDEM). Acad. Emerg. Med. 2017; 24: 328–36. 27743490 10.1111/acem.13118

[2] LaMantia MA , Platts‐Mills TF , Biese K *et al*. Predicting hospital admission and returns to the emergency department for elderly patients. Acad. Emerg. Med. 2010; 17: 252–9. 20370757 10.1111/j.1553-2712.2009.00675.x PMC5985811

[3] Ray P , Birolleau S , Lefort Y *et al*. Acute respiratory failure in the elderly: etiology, emergency diagnosis and prognosis. Crit. Care 2006; 10: 82. 10.1186/cc4926 PMC1550946 16723034

[4] Nehme Z , Bernard S , Cameron P *et al*. Using a cardiac arrest registry to measure the quality of emergency medical service care: decade of findings from the Victorian ambulance cardiac arrest registry. Circ. Cardiovasc. Qual. Outcomes 2015; 8: 56–66. 25604556 10.1161/CIRCOUTCOMES.114.001185

[5] Andrew E , Cox S , Smith K . Linking ambulance records with hospital and death index data to evaluate patient outcomes. Int. J. Gen. Med. 2022; 15: 567–72. 35046714 10.2147/IJGM.S328149 PMC8763257

[6] Prekker M , Feemster L , Hough C *et al*. The epidemiology and outcome of prehospital respiratory distress. Acad. Emerg. Med. 2014; 21: 543–50. 24842506 10.1111/acem.12380 PMC4247789

[7] Kauppi W , Herlitz J , Magnusson C , Palmer L , Axelsson C . Characteristics and outcomes of patients with dyspnoea as the main symptom, assessed by prehospital emergency nurses – a retrospective observational study. BMC Emerg. Med. 2020; 20: 1–11. 32859155 10.1186/s12873-020-00363-6 PMC7456019

[8] Kelly AM , Holdgate A , Keijzers G *et al*. Epidemiology, prehospital care and outcomes of patients arriving by ambulance with dyspnoea: an observational study. Scand. J. Trauma Resusc. Emerg. Med. 2016; 24: 113. 27658711 10.1186/s13049-016-0305-5 PMC5034604

[9] Hutchinson A , Pickering A , Williams P , Bland JM , Johnson MJ . Breathlessness and presentation to the emergency department: a survey and clinical record review. BMC Pulm. Med. 2017; 17: 53. 28320369 10.1186/s12890-017-0396-4 PMC5360046

[10] Ambulance Victoria . Clinical Practice Guidelines for Ambulance and MICA Paramedics. 2018. [Cited 27 Jul 2022.] Available from URL: https://www.ambulance.vic.gov.au/wp‐content/uploads/2018/07/Clinical‐Practice‐Guidelines‐2018‐Edition‐1.4.pdf

[11] Bernhard M , Lichtenstern C , Eckmann C , Weigand M . The early antibiotic therapy in septic patients – milestone or sticking point? Crit. Care 2014; 18: 671. 25672873 10.1186/s13054-014-0671-1 PMC4331420

[12] Pandor A , Thokala P , Goodacre S *et al*. Pre‐hospital non‐invasive ventilation for acute respiratory failure: a systematic review and cost‐effectiveness evaluation. Health Technol. Assess. 2015; 19: 101–2. 10.3310/hta19420 PMC4781299 26102313

