# Supplementary material for: Epidemiology, outcomes and predictors of mortality in patients transported by ambulance for dyspnoea: A population‐based cohort study
Source: Emerg Med Australas. 2022 Aug 2;35(1):48–55. doi: 10.1111/1742-6723.14053 (PMC10947453; doi:10.1111/1742-6723.14053)
Supplement: Supplementary file 1 — Appendix S1. Dataset linkage processes. [file EMM-35-48-s007.docx]

## **Appendix S1. Dataset linkage processes.**

For this study, data entered into the Ambulance Victoria electronic patient care record were linked to other relevant Victorian datasets, including:

1. Victorian Emergency Minimum Dataset (VEMD): Victorian Department of Health dataset comprising demographic, administrative and clinical data detailing emergency department (ED) presentations at public hospitals in the state. Data is submitted by individual health services and is then subject to validation checks. For this study, EMS patient identifiers were matched with Department of Health identifiers using Dataflux software with deterministic data linkage and fuzzy matching for variables such as names and dates. ED presentations for matched patients were then linked to ambulance cases as follows:
   1. Where the patient was transported to hospital by ambulance, the VEMD arrival time was required to be within one hour of the ambulance ED arrival time.
   2. Where the patient contacted ambulance but was not transported to hospital, the VEMD arrival time was required to be within 48 hours of the emergency call for ambulance. If multiple VEMD records existed within the 48-hour period, the presentation occurring closest in time to the ambulance call was used.
2. Victorian Admitted Episodes Dataset (VAED): Victorian Department of Health dataset comprising demographic, clinical and administrative data relating to each admitted episode of care occurring in public and private hospitals, as well as rehabilitation centres, extended care facilities and day procedure centres in the state. For this study, EMS patient identifiers were matched with Department of Health identifiers using Dataflux software with deterministic data linkage and fuzzy matching for variables such as names and dates. For matched patients, individual admitted episodes of care occurring up to 48 hours after the emergency ambulance call were linked to the ambulance patient care record data. Where multiple admitted episodes were recorded within the 48 hours, the episode occurring closest in time to the ambulance call was used.
3. Victorian Death Index (VDI): Victorian Department of Health dataset capturing the date and cause of all deaths in Victoria. For matched patients, death records were linked to all ambulance contacts occurring in the study period.

## Study definitions

Socio-economic status was determined using the Index of Relative Socio-Economic Disadvantage Score (IRSD), a validated measure that ranks individual post-codes into deciles of relative disadvantage. The score is derived from Census data and includes household income, education level, employment status, occupation, housing ownership, and non-English speaking background (1). For this analysis, we divided the IRSD into quintiles, with the 1^st^ quintile being the most disadvantaged (comprising of ISRD deciles 1 and 2) and the 5^th^ quintile being least disadvantaged (IRSD deciles 9 and 10).

Geographic remoteness was determined through the residential area postcode of each event using The Accessibility and Remoteness Index of Australia (ARIA) – a geographic accessibility index that divides Australia into five classes of remoteness (‘Major City’, ‘Inner Regional’, ‘Outer Regional’, ‘Remote’, and ‘Very Remote’) to reflect relative access to services in non-metropolitan Australia (2). Due to low numbers of patients from ‘remote’ or ‘very remote’ regions, these groups were combined with the ‘outer regional’ group for the purposes of this study.

Final hospital diagnoses were broadly categorised using the following International Classification of Diseases [ICD] 10 V9.0 criteria:

1. Cardiovascular diagnosis: I00-I99
2. Respiratory diagnosis: J00-J998
3. Infective diagnosis: A000-B99
4. Oncological diagnosis: C000-D899
5. Endocrine diagnosis: E000-E999
6. Mental health diagnosis: F000-F99
7. Neurological diagnosis: G00-G998
8. Gastrointestinal diagnosis: K000-K938
9. Injury or poisoning: S0000-Y98
10. Non-specific dyspnoea: R000-R99
11. Other diagnoses: H000-H959, L00-Q999, S001-Z999, Z000-Z999

Specific final diagnoses were defined according to the following ICD 10 V9.0 criteria:

1. Lower respiratory tract infection: J100, J110, J22, J851, J12-J18, J120-J189, J200-J219, B012, B052, B953
2. Chronic obstructive pulmonary disease: J431-J449, J982, J983
3. Asthma: J450-J46
4. Upper respiratory tract infection: J00-J069, J300-J420, J101, J108, J111, J118
5. Pleural effusion: J90-J91, J940-J942
6. Bronchiectasis: J47
7. Interstitial lung disease: J60-J64, J620-J678, J702, J703, J841-J848
8. Pneumothorax: J930-J939
9. Other respiratory: J00-J998 excluding codes categorised above
10. Heart failure: I110, I130, I132, I255, I420-422, I425-I438, I500-I509, U822
11. Atrial fibrillation: I480-I489
12. Other arrhythmia: I440-I479, I490-I499
13. Non-ST elevation myocardial infarction: I200, I214, I219
14. ST elevation myocardial infarction: I210-I213, I220-I229
15. Pulmonary embolism: I260, I269
16. Other cardiovascular: I00-I99 excluding codes categorised above
17. Sepsis: A021, A227, A267, A327, A427, B377, A400-A419, A4150-A4158, R651
18. Anaphylaxis or angioedema: T780-T783, T805, T886
19. Anxiety: F064, F4001, F408-F419
20. Non-specific dyspnoea: R000-R99

# References

1. Australian Bureau of Statistics. Census of Population and Housing: Socio-Economic Indexes for Areas (SEIFA), Australia, 2016. . Canberra: ABS Website; 2018.

2. Australian Bureau of Statistics. Australian Statistical Geography Standard (ASGS): Volume 1 - Main Structure and Greater Capital City Statistical Areas July 2016. . Canberra: ABS Website; 2016.
