# Supplementary figures and images for: Epidemiology, outcomes and predictors of mortality in patients transported by ambulance for dyspnoea: A population‐based cohort study
Source: Emerg Med Australas. 2022 Aug 2;35(1):48–55. doi: 10.1111/1742-6723.14053 (PMC10947453; doi:10.1111/1742-6723.14053)

**Figure S1. Cohort derivation.**

**
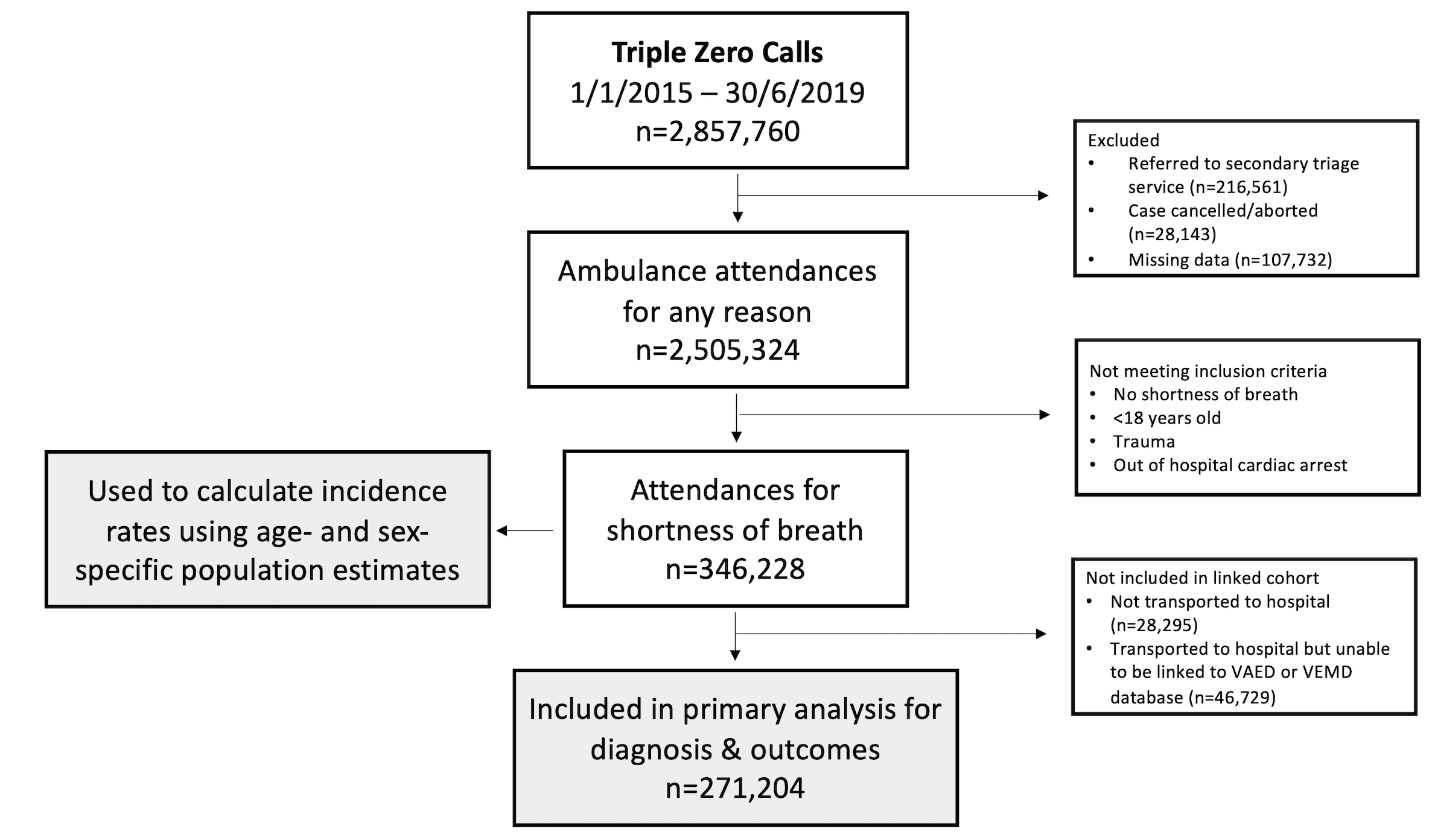
**

Supplement: Supplementary file 2 — Figure S1. Cohort derivation. [file EMM-35-48-s003.docx]
