# Supplementary material for: Epidemiology, outcomes and predictors of mortality in patients transported by ambulance for dyspnoea: A population‐based cohort study
Source: Emerg Med Australas. 2022 Aug 2;35(1):48–55. doi: 10.1111/1742-6723.14053 (PMC10947453; doi:10.1111/1742-6723.14053)
Supplement: Supplementary file 3 — Table S1. Incidence of shortness of breath. [file EMM-35-48-s011.docx]

**Table S1. Incidence of shortness of breath.**

|  | **Incidence per 100,000 person years (95% CI)** |
| --- | --- |
| **Overall** | 1,566 (1,561 – 1,571) |
|  |  |
| **Age**  18-24 years  25-29 years  30-34 years  35-39 years  40-44 years  45-49 years  50-54 years  55-59 years  60-64 years  65-69 years  70-74 years  75+ years | 367 (368 – 383)  341 (334 – 349)  411 (403 – 420)  510 (500 – 520)  635 (624 – 647)  786 (773 – 799)  911 (897 – 926)  1,151 (1,134 – 1,167)  1,547 (1,527 – 1,567)  2,167 (2,142 – 2,193)  3,404 (3,368 – 3,439)  8,400 (8,360 – 8,442) |
|  |  |
| **Sex**  Male  Female | 1,545 (1,538 – 1,553)  1,586 (1,578 – 1,593) |
|  |  |
| **Sex***  Male  Female | 1,635 (1,627 – 1,643)  1,516 (1,509 – 1,523) |
|  |  |
| **Region***  City  Inner regional  Outer regional & remote | 1,568 (1,563 – 1,574)  1,629 (1,617 – 1,642)  1,503 (1,478 – 1,528) |
|  |  |
| **Socioeconomic status***  Quintile 1 (lowest)  Quintile 2 (low)  Quintile 3 (middle)  Quintile 4 (high)  Quintile 5 (highest) | 2,269 (2,255 – 2,283)  1,912 (1,899 – 1,925)  1,520 (1,509 – 1,532)  1,317 (1,306 – 1,327)  889 (881 – 898) |
|  |  |
| **Year**  2015  2016  2017  2018  2019 | 1,552 (1,541 – 1,563)  1,546 (1,535 – 1,557)  1,610 (1,599 – 1,621)  1,539 (1,528 – 1,550  1,594 (1,579 – 1,610) |

*Age-adjusted incidence.
