# Supplementary material for: Epidemiology, outcomes and predictors of mortality in patients transported by ambulance for dyspnoea: A population‐based cohort study
Source: Emerg Med Australas. 2022 Aug 2;35(1):48–55. doi: 10.1111/1742-6723.14053 (PMC10947453; doi:10.1111/1742-6723.14053)
Supplement: Supplementary file 4 — Table S2. Differences in patient characteristics between those deemed by paramedics not to require transport and those transported to hospital. [file EMM-35-48-s005.docx]

**Table S2. Differences in patient characteristics between those deemed by paramedics not to require transport and those transported to hospital.**

|  | Not transported  N=17,687 | Transported  N=317,933 | Standardised difference* |
| --- | --- | --- | --- |
| Age (years) | 58 (37 - 77) | 73 (58 – 83) | -0.57 |
| Sex (female) | 10,670 (60.3%) | 162,894 (51.3%) | 0.18 |
| ARIA  Major City  Inner Regional  Outer Regional/Remote | 12,672 (72.6%)  3,944 (22.6%)  841 (4.8%) | 228,427 (72.5%)  70,250 (22.3%)  16,348 (5.1%) | 0.02 |
| Socio-economic status (IRSD)  Quintile 1 (lowest)  Quintile 2  Quintile 3  Quintile 4  Quintile 5 (highest) | 4,808 (30.4%)  3,336 (21.1%)  2,945 (18.6%)  2,756 (17.4%)  1,985 (12.6%) | 85,125 (29.5%)  63,542 (22.0%)  56,299 (19.5%)  49,258 (17.1%)  34,127 (11.8%) | 0.04 |
| Hypertension | 4,897 (27.9%) | 134,368 (42.7%) | 0.31 |
| Hyperlipidaemia | 2,922 (16.6%) | 83,815 (26.6%) | 0.24 |
| Diabetes mellitus | 2,231 (12.7%) | 68,502 (21.8%) | 0.24 |
| Chronic kidney disease | 306 (1.7%) | 17,658 (5.6%) | 0.21 |
| Prior coronary disease | 1,831 (10.4%) | 78,578 (25.0%) | 0.39 |
| Prior heart failure | 1,233 (7.0%) | 52,202 (16.6%) | 0.30 |
| Prior atrial fibrillation | 1,297 (7.4%) | 48,204 (15.3%) | 0.25 |
| COPD | 2,062 (11.7%) | 72,606 (23.1%) | 0.30 |
| Initial Respiratory Status  Normal respiratory status  Mild respiratory distress  Moderate respiratory distress  Severe respiratory distress  Depressed respirations  Apnoeic | 10,672 (76.4%)  2,674 (19.1%)  379 (2.7%)  113 (0.8%)  26 (0.2%)  48 (0.3%) | 120,939 (45.2%)  78,725 (30.1%)  39,722 (15.2%)  20,624 (7.9%)  392 (0.2%)  495 (0.2%) | 0.75 |
| Median respiratory rate (breaths/min) | 18 (16 – 22) | 24 (18 – 28) | -0.56 |
| Median SpO_2_ (%) | 98 (96 – 99) | 95 (90 – 98) | 0.59 |
| Febrile (T≥38.0) | 1,436 (8.7%) | 48,298 (16.1%) | 0.22 |
| Tachycardic (HR ≥100bpm) | 4,868 (27.7%) | 135,632 (42.9%) | 0.32 |
| Hypotensive (SBP <90mmHg) | 100 (0.6%) | 10,257 (3.3%) | 0.20 |
| Hypertension (SBP >180mmHg) | 361 (2.1%) | 21,809 (6.9%) | 0.24 |
| 30-day mortality | 554 (3.1%) | 25,890 (8.1%) | 0.22 |
| 1-year mortality | 1,657 (9.4%) | 67,506 (21.2%) | 0.33 |

*Standardised difference = difference in means or proportions divided by standard error; significant difference defined as absolute value greater than 0.10.
