# Supplementary material for: Epidemiology, outcomes and predictors of mortality in patients transported by ambulance for dyspnoea: A population‐based cohort study
Source: Emerg Med Australas. 2022 Aug 2;35(1):48–55. doi: 10.1111/1742-6723.14053 (PMC10947453; doi:10.1111/1742-6723.14053)
Supplement: Supplementary file 5 — Table S3. Differences in patient characteristics between the linked and unlinked cohorts. [file EMM-35-48-s001.docx]

**Table S3. Differences in patient characteristics between the linked and unlinked cohorts.**

|  | Not linked  n=46,673 | Linked  n=271,204 | Standardised difference* |
| --- | --- | --- | --- |
| Age | 72 (54-84) | 74 (59-83) | -0.10 |
| Sex (female) | 23,589 (50.4%) | 139,305 (51.4%) | 0.02 |
| ARIA  Major City  Inner Regional  Outer Regional/Remote | 30.013 (66.1%)  12,166 (26.8%)  3,230 (6.9%) | 198,414 (73.6%)  70,250 (21.5%)  13,118 (4.8%) | 0.17 |
| Socio-economic status (IRSD)  Quintile 1 (lowest)  Quintile 2  Quintile 3  Quintile 4  Quintile 5 (highest) | 12,568 (30.1%)  9,015 (21.6%)  7,923 (19.0%)  7,063 (16.9%)  5,192 (12.4%) | 72,557 (29.4%)  54,529 (22.1%)  48,376 (19.6%)  42,195 (17.1%)  28,935 (11.7%) | 0.03 |
| Hypertension | 17,730 (38.7%) | 116,638 (43.4%) | 0.09 |
| Hyperlipidaemia | 10,590 (23.1%) | 73,225 (27.2%) | 0.09 |
| Diabetes mellitus | 8,686 (19.0%) | 59,816 (22.2%) | 0.08 |
| Chronic kidney disease | 2,063 (4.4%) | 15,595 (5.8%) | 0.06 |
| Prior coronary disease | 9,927 (21.7%) | 68,651 (25.5%) | 0.09 |
| Prior heart failure | 6,470 (14.1%) | 45,732 (17.0%) | 0.08 |
| Prior atrial fibrillation | 6,081 (13.3%) | 42,123 (15.7%) | 0.07 |
| COPD | 8,781 (19.2%) | 63,825 (23.7%) | 0.11 |
| Initial Respiratory Status  Normal respiratory status  Mild respiratory distress  Moderate respiratory distress  Severe respiratory distress  Depressed respirations  Apnoeic | 18,170 (47.7%)  11,164 (29.3%)  5,241 (13.8%)  3,034 (6.5%)  83 (0.2%)  169 (0.4%) | 102,769 (46.0%)  67,561 (30.1%)  34,481 (15.2%)  17,590 (7.9%)  309 (0.1%)  326 (0.2%) | 0.10 |
| Median respiratory rate (breaths/min) | 24 (18-28) | 22 (18-28) | -0.07 |
| Median SpO_2_ (%) | 95 (90-98) | 96 (91-98) | 0.04 |
| Febrile (T≥38.0) | 6,702 (15.4%) | 41,596 (16.2%) | 0.02 |
| Tachycardic (HR ≥100bpm) | 19,763 (42.7%) | 115,869 (42.9%) | 0.004 |
| Hypotensive (SBP <90mmHg) | 1,704 (3.7%) | 8,553 (3.2%) | 0.03 |
| Hypertension (SBP>180mmHg) | 3,138 (6.8%) | 18,571 (6.9%) | 0.004 |
| 30-day mortality | 1,607 (3.4%) | 24,283 (9.0%) | 0.23 |
| 1-year mortality | 3,573 (7.7%) | 63,945 (23.6%) | 0.44 |

*Standardised difference = difference in means or proportions divided by standard error; significant difference defined as absolute value greater than 0.10.
