# Supplementary material for: Epidemiology, outcomes and predictors of mortality in patients transported by ambulance for dyspnoea: A population‐based cohort study
Source: Emerg Med Australas. 2022 Aug 2;35(1):48–55. doi: 10.1111/1742-6723.14053 (PMC10947453; doi:10.1111/1742-6723.14053)
Supplement: Supplementary file 6 — Table S4. Ambulance and hospital management details. [file EMM-35-48-s004.docx]

**Table S4. Ambulance and hospital management details.**

|  | 18-49 years  n=42,638 | 50-74 years  n=98,119 | 75+ years  n=130,402 | Overall  n=271,204 |  |
| --- | --- | --- | --- | --- | --- |
| Ambulance data | | | | | |
| Time to EMS arrival (mins) | 11 (8-16) | 11 (8-17) | 12 (9-20) | 11 (8-18) |  |
| Time at location (mins) | 16 (11-23) | 19 (13-25) | 20 (15-27) | 19 (14-26) |  |
| Time in transit (mins) | 19 (12-28) | 20 (12-30) | 19 (12-28) | 19 (12-28) |  |
| Time at hospital (mins) | 46 (35-59) | 47 (36-60) | 48 (37-60) | 47 (37-60) |  |
| Total case time (mins) | 99 (80-121) | 105 (86-128) | 107 (89-130) | 105 (86-128) |  |
| Transport priority |  |  |  |  |  |
| Lights and sirens | 5,715 (13.6%) | 14,524 (15.0%) | 16,677 (13.0%) | 36,915 (13.8%) |  |
| Urgent/acute | 33,629 (80.2%) | 77,058 (79.8%) | 102,128 (79.7%) | 212,815 (79.8%) |  |
| Non-urgent | 2,426 (5.7%) | 4500 (4.7%) | 7,982 (6.2%) | 14,908 (5.6%) |  |
| Pre-hospital therapy |  |  |  |  |  |
| Oxygen | 9,415 (22.1%) | 37,984 (38.7%) | 59,740 (45.8%) | 107,139 (39.5%) |  |
| Non-invasive ventilation | 150 (0.4%) | 2,307 (2.4%) | 3,763 (2.9%) | 6,220 (2.3%) |  |
| Intubation | 256 (0.6%) | 339 (0.4%) | 199 (0.2%) | 794 (0.3%) |  |
| Inhaled bronchodilators | 6,526 (15.3%) | 21,108 (21.5%) | 19,144 (14.7%) | 46,778 (17.3%) |  |
| Adrenaline | 2,037 (4.7%) | 1,792 (1.8%) | 791 (0.6%) | 4,620 (1.7%) |  |
| Corticosteroids | 1,638 (3.8%) | 7,405 (7.6%) | 5,862 (4.5%) | 14,905 (5.5%) |  |
| Aspirin | 5,023 (11.8%) | 13,700 (14.0%) | 12,645 (9.7%) | 31,368 (11.6%) |  |
| Heparin | 142 (0.3%) | 614 (0.6%) | 257 (0.2%) | 1,013 (0.4%) |  |
| Glyceryl trinitrate | 4,826 (11.3%) | 15,984 (16.3%) | 18,723 (14.4%) | 39,533 (14.6%) |  |
| IV fluids | 4,801 (11.3%) | 10,576 (10.8%) | 9,409 (7.2%) | 24,786 (9.1%) |  |
| Frusemide | 25 (0.06%) | 494 (0.5%) | 1,388 (1.1%) | 1,907 (0.9%) |  |
| Antibiotics | 13 (0.03%) | 56 (0.06%) | 31 (0.02%) | 100 (0.04%) |  |
| Transported to public hospital | 41,539 (97.4%) | 92,488 (94.3%) | 115,967 (88.9%) | 249,994 (92.2%) |  |
|  | | | | | |
| Hospital data | | | | | |
| Time in ED (hours) | 3.7 (2.5-5.9) | 4.9 (3.3-13.5) | 5.6 (3.7-8.9) | 4.9 (3.3-8.2) |  |
| Discharged from ED | 17,598 (42.3%) | 19,144 (20.9%) | 13,603 (11.9%) | 50,345 (20.4%) |  |
| Admitted to hospital | 23,937 (57.5%) | 71,913 (78.6%) | 99,502 (87.2%) | 195,357 (79.0%) |  |
| Length of hospital stay (days) | 1 (1-3) | 3 (1-5) | 3 (1-7) | 3 (1-6) |  |
| ICU admission | 3,492 (14.1%) | 11,303 (14.6%) | 9,473 (8.3%) | 24,268 (11.2%) |  |
| Length of ICU stay (hours) | 47 (24-91) | 52 (27-99) | 48 (24-86) | 50 (25-93) |  |
| Non-invasive ventilation | 1,624 (3.8%) | 7,939 (8.1%) | 8,218 (6.3%) | 17,781 (6.6%) |  |
| Mechanical ventilation | 1,102 (2.6%) | 1,130 (2.2%) | 887 (0.7%) | 4,119 (1.5%) |  |
