# Supplementary material for: Epidemiology, outcomes and predictors of mortality in patients transported by ambulance for dyspnoea: A population‐based cohort study
Source: Emerg Med Australas. 2022 Aug 2;35(1):48–55. doi: 10.1111/1742-6723.14053 (PMC10947453; doi:10.1111/1742-6723.14053)
Supplement: Supplementary file 7 — Table S5. Pre‐hospital interventions administered by EMS and concordance with final hospital diagnosis. [file EMM-35-48-s008.docx]

**Table S5. Pre-hospital interventions administered by EMS and concordance with final hospital diagnosis.**

|  | **Pre-hospital interventions administered by EMS by final hospital diagnosis** | | | | | | | | |
| --- | --- | --- | --- | --- | --- | --- | --- | --- | --- |
| **Final hospital diagnosis** | **NIV** | **Inhaled broncho-dilator** | **Cortico-steroid** | **Aspirin** | **Heparin** | **Glyceryl trinitrate** | **Frusemide** | **IV Fluid** | **Anti-biotics** |
| **LRTI** | 0.5% | 14.6% | 3.8% | 3.9% | 0.0% | 5.9% | 0.5% | 7.7% | 0.07% |
| **COPD** | 4.4% | 60.5% | 23.1% | 2.6% | 0.0% | 4.3% | 0.4% | 5.3% | 0.02% |
| **Asthma** | 0.6% | 69.6% | 15.7% | 1.4% | 0.0% | 1.8% | 0.2% | 4.6% | 0.00% |
| **URTI** | 0.6% | 13.9% | 3.2% | 2.9% | 0.0% | 3.0% | 0.1% | 6.9% | 0.04% |
| **Heart Failure** | 8.5% | 10.0% | 2.6% | 11.3% | 0.1% | 28.7% | 4.1% | 4.0% | 0.00% |
| **STEMI** | 3.2% | 2.8% | 0.9% | 66.9% | 34.3% | 39.6% | 1.3% | 42.4% | 0.00% |
| **NSTEACS** | 5.2% | 7.5% | 2.4% | 41.1% | 1.0% | 55.1% | 2.3% | 12.4% | 0.00% |
| **Non-specific SOB** | 0.3% | 5.1% | 1.3% | 24.7% | 0.1% | 29.0% | 0.1% | 8.0% | 0.01% |
| **Infection** | 1.3% | 7.9% | 1.7% | 3.7% | 0.0% | 3.7% | 0.2% | 18.6% | 0.30% |
| **Injury or poisoning** | 0.5% | 10.0% | 5.6% | 3.2% | 0.0% | 3.5% | 0.1% | 17.8% | 0.01% |

COPD indicates chronic obstructive pulmonary disease; EMS, emergency medical services; IV, intravenous; LRTI, lower respiratory tract infection; NIV, non-invasive ventilation; NSTEACS, Non-ST-elevation acute coronary syndrome; SOB, shortness of breath; STEMI, ST-elevation myocardial infarction; and URTI, upper respiratory tract infection.
