# Supplementary material for: Epidemiology, outcomes and predictors of mortality in patients transported by ambulance for dyspnoea: A population‐based cohort study
Source: Emerg Med Australas. 2022 Aug 2;35(1):48–55. doi: 10.1111/1742-6723.14053 (PMC10947453; doi:10.1111/1742-6723.14053)
Supplement: Supplementary file 8 — Table S6. Differences in patient characteristics between those discharged versus admitted from the ED. [file EMM-35-48-s010.docx]

**Table S6. Differences in patient characteristics between those discharged versus admitted from the ED.**

|  | Discharged  N=50,346 | Admitted  N=195,357 | Standardised difference* |
| --- | --- | --- | --- |
| Age (years) | 60 (43 – 76) | 75 (62 – 84) | -0.67 |
| Sex (female) | 27,730 (55.1%) | 98,000 (50.2%) | 0.10 |
| ARIA  Major City  Inner Regional  Outer Regional/Remote | 32,996 (66.3%)  13,383 (26.9%)  3,410 (6.8%) | 148,183 (76.2%)  38,387 (19.7%)  7,824 (4.0%) | 0.23 |
| Socio-economic status (IRSD)  Quintile 1 (lowest)  Quintile 2  Quintile 3  Quintile 4  Quintile 5 (highest) | 14,991 (33.3%)  10,359 (23.0%)  8,504 (18.9%)  7,010 (15.6%)  4,195 (9.3%) | 52,799 (29.7%)  39,817 (22.4%)  35,235 (19.9%)  30,245 (17.0%)  19,473 (11.0%) | 0.09 |
| Hypertension | 16,475 (33.1%) | 88,370 (45.6%) | 0.26 |
| Hyperlipidaemia | 10,540 (21.2%) | 55,790 (28.8%) | 0.18 |
| Diabetes mellitus | 7,961 (16.0%) | 46,833 (25.2%) | 0.20 |
| Chronic kidney disease | 1,461 (2.9%) | 12,707 (6.6%) | 0.17 |
| Prior coronary disease | 9,439 (19.0%) | 51,937 (26.8%) | 0.19 |
| Prior heart failure | 4,250 (8.5%) | 36,251 (18.7%) | 0.30 |
| Prior atrial fibrillation | 4,745 (9.5%) | 32,138 (16.6%) | 0.21 |
| COPD | 8,329 (16.7%) | 49,789 (25.7%) | 0.22 |
| Initial Respiratory Status  Normal respiratory status  Mild respiratory distress  Moderate respiratory distress  Severe respiratory distress  Depressed respirations  Apnoeic | 25,209 (60.8%)  11,118 (26.8%)  3,680 (8.9%)  1,228 (3.0%)  45 (0.1%)  93 (0.2%) | 68,658 (42.5%)  49,722 (30.8%)  27,689 (17.1%)  14,773 (9.1%)  235 (0.1%)  206 (0.1%) | 0.44 |
| Median respiratory rate (breaths/min) | 20 (18 – 24) | 24 (20 – 30) | -0.40 |
| Median SpO_2_ (%) | 97 (95 – 98) | 94 (89 – 97) | 0.55 |
| Febrile (T≥38.0) | 3,272 (7.0%) | 33,594 (18.1%) | 0.34 |
| Tachycardic (HR ≥100bpm) | 18,679 (37.2%) | 87,136 (44.7%) | 0.15 |
| Hypotensive (SBP <90mmHg) | 687 (1.4%) | 6,812 (3.5%) | 0.13 |
| Hypertension (SBP >180mmHg) | 16,475 (33.1%) | 88,370 (45.6%) | 0.26 |
| 30-day mortality | 1,093 (2.2%) | 18,772 (9.6%) | 0.32 |
| 1-year mortality | 4,912 (9.8%) | 50,196 (25.7%) | 0.43 |

*Standardised difference = difference in means or proportions divided by standard error; significant difference defined as absolute value greater than 0.10.
