# Supplementary material for: Epidemiology, outcomes and predictors of mortality in patients transported by ambulance for dyspnoea: A population‐based cohort study
Source: Emerg Med Australas. 2022 Aug 2;35(1):48–55. doi: 10.1111/1742-6723.14053 (PMC10947453; doi:10.1111/1742-6723.14053)
Supplement: Supplementary file 9 — Table S7. Final hospital diagnoses stratified by sex and age group. [file EMM-35-48-s009.docx]

**Table S7. Final hospital diagnoses stratified by sex and age group.**

|  | **Male** | | | **Female** | | | **All patients** |
| --- | --- | --- | --- | --- | --- | --- | --- |
|  | **18-49 years**  **n=17,730** | **50-74 years**  **n=51,489** | **75+ years**  **n=62,635** | **18-49 years**  **n=24,937** | **50-74 years**  **n=46,619** | **75+ years**  **n=67,749** | **All ages**  **n=271,204** |
| **Respiratory** | **4,251 (24.0%)** | **18,361 (35.7%)** | **25,833 (41.2%)** | **7,592 (30.4%)** | **18,841 (40.4%)** | **25,383 (37.5%)** | **100,269 (37.0%)** |
| LRTI | 1,347 (7.6%) | 5,561 (10.8%) | 11,173 (17.8%) | 1,868 (7.5%) | 5,156 (11.1%) | 11,303 (16.7%) | 36,411 (13.4%) |
| COPD exacerbation | 419 (2.4%) | 8,430 (16.4%) | 9,099 (14.5%) | 800 (3.2%) | 8,456 (18.1%) | 7,746 (11.4%) | 34,953 (12.9%) |
| Asthma exacerbation | 1,280 (7.2%) | 888 (1.7%) | 306 (0.5%) | 3,282 (13.2%) | 1,940 (4.2%) | 1,090 (1.6%) | 8,787 (3.2%) |
| URTI | 582 (3.3%) | 998 (1.9%) | 1,159 (1.9%) | 1,099 (4.4%) | 1,244 (2.7%) | 1,718 (2.5%) | 6,800 (2.5%) |
| Pleural effusion | 29 (0.2%) | 272 (0.5%) | 488 (0.8%) | 32 (0.1%) | 208 (0.5%) | 388 (0.6%) | 1,417 (0.5%) |
| Bronchiectasis | 27 (0.2%) | 105 (0.2%) | 242 (0.4%) | 33 (0.1%) | 278 (0.6%) | 526 (0.8%) | 1,211 (0.5%) |
| ILD exacerbation | 7 (0.04%) | 198 (0.4%) | 436 (0.7%) | 6 (0.02%) | 139 (0.3%) | 264 (0.4%) | 1,045 (0.4%) |
| Pneumothorax | 103 (0.6%) | 157 (0.3%) | 132 (0.2%) | 57 (0.2%) | 71 (0.2%) | 53 (0.1%) | 574 (0.2%) |
|  |  |  |  |  |  |  |  |
| **Cardiovascular** | **1,883 (10.6%)** | **10,889 (21.2%)** | **15,377 (24.6%)** | **1,685 (6.8%)** | **7,611 (16.3%)** | **18,975 (28.0%)** | **56,530 (20.8%)** |
| Heart failure | 257 (1.5%) | 3,146 (6.1%) | 8,564 (13.7%) | 168 (0.7%) | 2,304 (4.9%) | 10,245 (15.1%) | 24,686 (9.1%) |
| Atrial fibrillation | 233 (1.3%) | 1,360 (2.6%) | 1,107 (1.8%) | 124 (0.5%) | 1,406 (3.0%) | 2,461 (3.6%) | 6,692 (2.5%) |
| Other arrhythmia | 350 (2.0%) | 786 (1.5%) | 593 (1.0%) | 659 (2.6%) | 769 (1.7%) | 769 (1.1%) | 3,928 (1.5%) |
| NSTEACS | 232 (1.3%) | 2,210 (4.3%) | 2,420 (3.9%) | 120 (0.5%) | 1,100 (2.4%) | 2,271 (3.4%) | 8,357 (3.1%) |
| STEMI | 209 (1.2%) | 951 (1.9%) | 366 (0.6%) | 38 (0.2%) | 320 (0.7%) | 355 (0.5%) | 2,239 (0.8%) |
| Pulmonary embolism | 124 (0.7%) | 560 (1.1%) | 377 (0.6%) | 220 (0.9%) | 536 (1.2%) | 596 (0.9%) | 2,413 (0.9%) |
|  |  |  |  |  |  |  |  |
| **Non-specific SOB** | **5,177 (29.2%)** | **9,988 (19.4%)** | **8.048 (12.9%)** | **6,530 (26.2%)** | **9,521 (20.4%)** | **9,646 (14.3%)** | **48,920 (18.0%)** |
| **Infective** | **683 (3.9%)** | **2,267 (4.4%)** | **3,305 (5.3%)** | **981 (3.9%)** | **1,971 (4.2%)** | **3,115 (4.6%)** | **12.324 (4.5%)** |
| Sepsis | 209 (1.2%) | 1,435 (2.8%) | 2,509 (4.0%) | 185 (0.7%) | 1,037 (2.2%) | 2,082 (3.1%) | 7,459 (2.8%) |
| **Poisoning or injury** | **1,579 (8.9%)** | **1,581 (3.1%)** | **1,281 (2.1%)** | **2,137 (8.6%)** | **1,578 (3.4%)** | **1,224 (1.8%)** | **9,383 (3.5%)** |
| Anaphylaxis | 307 (1.7%) | 177 (0.3%) | 37 (0.1%) | 811 (3.3%) | 372 (0.8%) | 83 (0.1%) | 1,787 (0.7%) |
| **Gastrointestinal** | **743 (4.2%)** | **1,977 (3.8%)** | **2,011 (3.2%)** | **908 (3.6%)** | **1,523 (3.3%)** | **4,479 (3.2%)** | **9,212 (3.4%)** |
| **Oncological** | **183 (1.0%)** | **1,849 (3.6%)** | **2,241 (3.6%)** | **273 (1.1%)** | **1,461 (3.1%)** | **1,684 (2.5%)** | **7,694 (2.8%)** |
| **Mental health** | **1,051 (5.9%)** | **770 (1.5%)** | **770 (1.2%)** | **1,321 (5.3%)** | **758 (1.6%)** | **954 (1.4%)** | **5,628 (2.1%)** |
| Anxiety | 365 (2.1%) | 276 (0.5%) | 175 (0.3%) | 676 (2.7%) | 425 (0.9%) | 382 (0.6%) | 2,299 (0.9%) |
| **Rheumatological** | **318 (1.8%)** | **709 (1.4%)** | **701 (1.1%)** | **565 (2.3%)** | **713 (1.5%)** | **999 (1.5%)** | **4,006 (1.5%)** |
| **Endocrine** | **401 (2.3%)** | **778 (1.5%)** | **767 (1.2%)** | **452 (1.8%)** | **614 (1.3%)** | **928 (1.4%)** | **3,940 (1.5%)** |
| **Neurological** | **355 (2.0%)** | **492 (1.0%)** | **374 (0.6%)** | **368 (1.5%)** | **370 (0.8%)** | **390 (0.6%)** | **2,349 (0.9%)** |

COPD indicates chronic obstructive pulmonary disease; ILD, interstitial lung disease, LRTI, lower respiratory tract infection; NSTEACS, non ST-elevation myocardial infarction; STEMI, ST elevation myocardial infarction; URTI, upper respiratory tract infection.
