# Supplementary material for: Epidemiology, outcomes and predictors of mortality in patients transported by ambulance for dyspnoea: A population‐based cohort study
Source: Emerg Med Australas. 2022 Aug 2;35(1):48–55. doi: 10.1111/1742-6723.14053 (PMC10947453; doi:10.1111/1742-6723.14053)
Supplement: Supplementary file 10 — Table S8. Mortality among patients transported by EMS. [file EMM-35-48-s006.docx]

**Table S8. Mortality among patients transported by EMS.**

|  | 18-49 years  n=42,638 | 50-74 years  n=98,119 | 75+ years  n=130,402 | Overall  n=271,204 |
| --- | --- | --- | --- | --- |
| 30-day mortality | 697 (1.6%) | 6,537 (6.7%) | 17,049 (13.1%) | 24,283 (9.0%) |
| If discharged from ED | 55 (0.3%) | 319 (1.7%) | 719 (5.3%) | 1,093 (2.2%) |
| If admitted to hospital | 531 (2.2%) | 5,183 (7.2%) | 13,058 (13.1%) | 18,772 (9.6%) |
| If requiring ICU admission | 205 (5.9%) | 1,370 (12.1%) | 1,504 (15.9%) | 3,079 (12.7%) |
| 1-year mortality | 1,970 (4.6%) | 17,874 (18.2%) | 44,104 (33.8%) | 63,948 (23.6%) |
