# Supplementary material for: Epidemiology, outcomes and predictors of mortality in patients transported by ambulance for dyspnoea: A population‐based cohort study
Source: Emerg Med Australas. 2022 Aug 2;35(1):48–55. doi: 10.1111/1742-6723.14053 (PMC10947453; doi:10.1111/1742-6723.14053)
Supplement: Supplementary file 11 — Table S9. 30‐day mortality rates for each diagnosis stratified by sex and age group. [file EMM-35-48-s002.docx]

**Table S9. 30-day mortality rates for each diagnosis stratified by sex and age group.**

|  | **Male** | | | **Female** | | | **All patients** |
| --- | --- | --- | --- | --- | --- | --- | --- |
|  | **18-49 years**  **n=17,730** | **50-74 years**  **n=51,489** | **75+ years**  **n=62,635** | **18-49 years**  **n=24,937** | **50-74 years**  **n=46,619** | **75+ years**  **n=67,749** | **All ages**  **n=271,204** |
| **Respiratory** | **2.2%** | **7.1%** | **14.4%** | **1.1%** | **5.8%** | **12.6%** | **9.5%** |
| LRTI | 2.8% | 7.5% | 14.6% | 1.7% | 6.0% | 13.6% | 10.9% |
| COPD exacerbation | 2.2% | 4.8% | 8.4% | 1.1% | 5.0% | 7.9% | 6.4% |
| Asthma exacerbation | 0.2% | 0.7% | 2.0% | 0.2% | 0.4% | 1.1% | 0.5% |
| URTI | 0.3% | 1.6% | 3.5% | 0.0% | 1.1% | 3.1% | 1.8% |
| Pleural effusion | 3.5% | 12.9% | 16.4% | 6.3% | 11.5% | 19.3% | 15.3% |
| Bronchiectasis | 0.0% | 0.0% | 5.4% | 0.0% | 3.6% | 7.4% | 5.1% |
| ILD exacerbation | 28.6% | 18.1% | 27.5% | 16.7% | 16.6% | 17.4% | 21.8% |
| Pneumothorax | 1.0% | 5.1% | 13.7% | 0.0% | 4.2% | 15.1% | 6.6% |
|  |  |  |  |  |  |  |  |
| **Cardiovascular** | **3.8%** | **6.4%** | **12.6%** | **3.1%** | **4.9%** | **11.7%** | **9.5%** |
| Heart failure | 5.1% | 7.3% | 11.8% | 1.8% | 4.4% | 10.4% | 9.8% |
| Atrial fibrillation | 0.0% | 1.7% | 5.2% | 0.0% | 1.2% | 3.9% | 2.9% |
| Other arrhythmia | 5.1% | 8.3% | 10.6% | 1.8% | 3.5% |  | 6.9% |
| NSTEACS | 1.3% | 3.4% | 12.2% | 0.8% | 3.2% | 12.3% | 8.3% |
| STEMI | 1.4% | 6.2% | 30.6% | 2.6% | 6.6% | 35.2% | 14.3% |
| Pulmonary embolism | 3.2% | 8.2% | 11.7% | 6.4% | 7.8% | 11.2% | 9.0% |
|  |  |  |  |  |  |  |  |
| **Non-specific SOB** | **0.8%** | **2.8%** | **7.0%** | **0.3%** | **2.4%** | **6.4%** | **3.6%** |
| **Infective** | **3.7%** | **13.5%** | **23.8%** | **2.0%** | **10.6%** | **24.2%** | **17.1%** |
| Sepsis | 11.5% | 19.2% | 28.8% | 10.8% | 19.0% | 33.0% | 25.8% |
| **Injury or poisoning** | **1.1%** | **4.2%** | **10.8%** | **0.7%** | **3.1%** | **8.1%** | **4.1%** |
| Anaphylaxis | 0% | 0% | 2.7% | 0% | 0% | 0% | 0.06% |
| **Gastrointestinal** | **3.2%** | **7.8%** | **13.7%** | **1.1%** | **6.0%** | **15.6%** | **9.5%** |
| **Oncological** | **38.2%** | **41.5%** | **35.6%** | **33.7%** | **37.2%** | **30.6%** | **36.2%** |
| **Mental health** | **0.7%** | **3.3%** | **12.0%** | **0.2%** | **1.7%** | **9.0%** | **4.0%** |
| Anxiety | 0.3% | 1.8% | 4.6% | 0.0% | 0.9% | 3.1% | 1.7% |
| **Rheumatological** | **1.3%** | **3.1%** | **6.6%** | **0.4%** | **2.2%** | **4.3%** | **3.3%** |
| **Endocrine** | **2.7%** | **6.2%** | **15.7%** | **1.1%** | **6.5%** | **12.8%** | **8.7%** |
| **Neurological** | **2.5%** | **8.5%** | **15.0%** | **0.5%** | **6.5%** | **7.7%** | **7.0%** |

COPD indicates chronic obstructive pulmonary disease; ILD, interstitial lung disease, LRTI, lower respiratory tract infection; NSTEACS, non ST-elevation myocardial infarction; STEMI, ST elevation myocardial infarction; URTI, upper respiratory tract infection.
